# Post-traumatic spinal hematoma in diffuse idiopathic skeletal hyperostosis (DISH)

**DOI:** 10.1007/s00330-023-09866-9

**Published:** 2023-06-29

**Authors:** Riku M. Vierunen, Ville V. Haapamäki, Mika P. Koivikko, Frank V. Bensch

**Affiliations:** Department of Radiology, HUS Diagnostic Center, University of Helsinki and Helsinki University Hospital, Haartmaninkatu 4, 00029 Helsinki, Finland

**Keywords:** Ankylosis, Hematoma, epidural, spinal, Hyperostosis, diffuse idiopathic skeletal, Magnetic resonance imaging, Spinal fractures

## Abstract

**Objectives:**

To determine the incidence of spinal hematoma and its relation to neurological deficit after trauma in patients with spinal ankylosis from diffuse idiopathic skeletal hyperostosis (DISH).

**Materials and methods:**

A retrospective review of 2256 urgent or emergency MRI referrals over a period of 8 years and nine months revealed 70 DISH patients who underwent CT and MRI scans of the spine. Spinal hematoma was the primary outcome. Additional variables were spinal cord impingement, spinal cord injury (SCI), trauma mechanism, fracture type, spinal canal narrowing, treatment type, and Frankel grades during injury, before and after treatment. Two trauma radiologists reviewed MRI scans blinded to initial reports.

**Results:**

Of 70 post-traumatic patients (54 men, median age 73, IQR 66–81) with ankylosis of the spine from DISH, 34 (49%) had spinal epidural hematoma (SEH) and 3 (4%) had spinal subdural hematoma, 47 (67%) had spinal cord impingement, and 43 (61%) had SCI. Ground-level fall (69%) was the most common trauma mechanism. A transverse, AO classification type B spine fracture (39%) through the vertebral body was the most common injury type. Spinal canal narrowing (*p* < .001) correlated and spinal cord impingement (*p* = .004) associated with Frankel grade before treatment. Of 34 patients with SEH, one, treated conservatively, developed SCI.

**Conclusions:**

SEH is a common complication after low-energy trauma in patients with spinal ankylosis from DISH. SEH causing spinal cord impingement may progress to SCI if not treated by decompression.

**Clinical relevance statement:**

Low-energy trauma may cause unstable spinal fractures in patients with spinal ankylosis caused by DISH. The diagnosis of spinal cord impingement or injury requires MRI, especially for the exclusion of spinal hematoma requiring surgical evacuation.

**Key Points:**

*• Spinal epidural hematoma is a common complication in post-traumatic patients with spinal ankylosis from DISH.*

*• Most fractures and associated spinal hematomas in patients with spinal ankylosis from DISH result from low-energy trauma.*

*• Spinal hematoma can cause spinal cord impingement, which may lead to SCI if not treated by decompression.*

## Introduction

Diffuse idiopathic skeletal hyperostosis (DISH) is a common systemic disorder of new bone formation and is associated with obesity, atherosclerosis, type 2 diabetes, male sex, and old age [1, 2]. New bone formation eventually leads to ankylosis mainly located in the thoracic but also in the cervical spine, which predisposes patients to unstable fractures and spinal epidural hematoma (SEH) even from low-energy trauma such as a ground-level fall (GLF) as typically seen in the elderly [1, 3, 4].

As a rare entity in the general population, SEH occurs spontaneously with an incidence of 1 per million per year [5]. Secondary to injury, spinal hematoma originates most likely from the epidural venous plexus or the damaged bone [6]. MRI is the most appropriate modality for the diagnosis of spinal hematoma but requires an experienced radiologist for a reliable diagnosis [6, 7]. Without timely diagnosis and treatment, post-traumatic SEH can cause spinal cord impingement and within hours may develop spinal cord injury (SCI) leading to possible permanent neurological impairment [8]. Spinal subdural hematoma (SSH) is much less common and occurs in the potential space between the dura and the arachnoid mater [9].

The original radiologic criteria define DISH as the presence of “flowing” osteophytes along the anterolateral aspects of at least four adjacent vertebrae, relative preservation of intervertebral disc height, absence of degenerative changes, and absence of ankylosis or erosions in the apophyseal or sacro-iliac joints [10]. New aspects challenge the original criteria as recent literature proposes a wider perspective for diagnosis in the clinical setting. Small pointy osteophytes in the thoracic spine without complete ankylosis suggest early-stage DISH [11], being obviously more readily visible on CT scans than radiographs. Improved detection of degenerative changes on CT complicates differential diagnosis of DISH [12]. DISH osteophytes tend to face anterolaterally to the right, i.e., away from the pulsating aorta, as confirmed by findings in patients with situs inversus totalis [13].

Several case reports and even large series of post-traumatic patients with ankylosed spines describe SEHs in separate or combined groups, or even without distinction of DISH and ankylosing spondylitis [4, 14–24]. Those studies lack blinded retrospective review by radiologists focusing on spinal hematoma, spinal cord impingement, SCI, and distinction from other ankylosing spinal disorders. The purpose of this study is to determine the incidence of spinal hematoma, major trauma mechanisms, and correlation to neurological deficits in spinal trauma with ankylosis from DISH.

## Materials and methods

The University Hospital’s institutional review board approved this retrospective study. Töölö Hospital, a part of Helsinki University Hospital, is the only level-1 trauma center for a catchment area of 1.67 million people, with routine imaging of whole-body and spinal CT and MRI for blunt trauma.

## Patient cohort, inclusion, and exclusion criteria

**Fig. 1:**
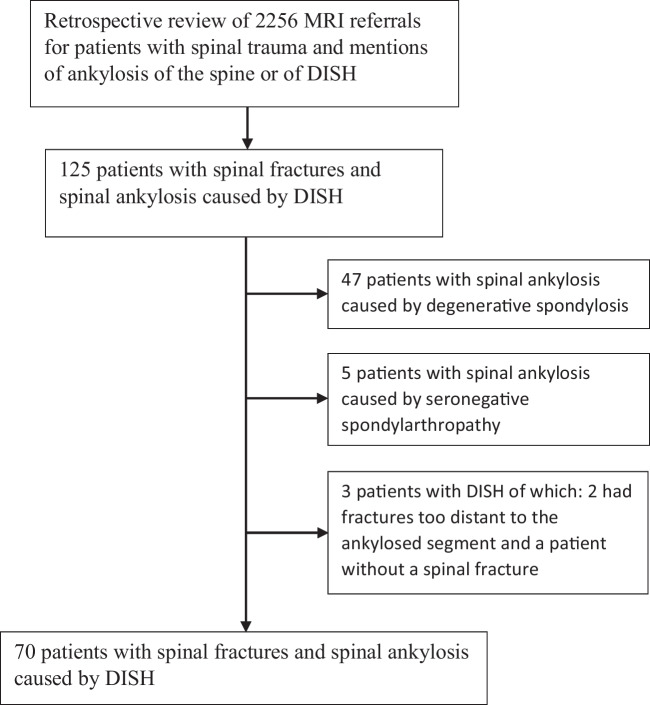
Flow chart showing the selection of study patients

A manual retrospective review covering a period of 8 years and nine months between January 2011 and September 2019 using Impax Picture Archiving and Communications System (Impax 6, Agfa Healthcare NV) revealed 2256 urgent or emergency MRI referrals. Patients over the age of 15 with mentions of trauma and ankylosis of the spine or DISH as well as both spinal MRI and a preceding CT scan, a spinal fracture at the level of or adjacent to the ankylosed segment, and aorta-evading syndesmophytes visible on CT along the anterior longitudinal ligament over at least four adjacent vertebrae were included [10, 11, 13]. Patients with radiological features of both DISH and seronegative spondylarthropathy (SpA), proven diagnosis of SpA in the medical records, ankylosis of the apophyseal joints, and those with fully ankylosed spines typical for ankylosing spondylitis were excluded. Also, patients with spinal ankylosis from degenerative spondylosis lacking anterolateral protrusion of syndesmophytes were excluded [3]. The reassessment of 2256 referrals produced a cohort of 125 patients with spinal fractures and spinal ankylosis, of which 70 had DISH (Fig. 1).

## Imaging

CT scans were acquired using a 64-slice CT scanner (Discovery CT 750 HD, GE Healthcare) with 2 mm coronal, sagittal, and axial reformats from the basic volumetric dataset of 0.625 mm slice thickness.

MRI scans were acquired using a 1.5-T closed-bore MRI scanner (Signa LX 1.5 T, GE Healthcare). MRI scans for spinal trauma are a secondary imaging modality and are imaged upon request. The trauma protocol consists of T2-, T1-, and short tau inversion recovery (STIR) images in the sagittal and T2W- and T1W- images in the transverse plane. Additional sequences are ordered if needed by the attending radiologist.

## Variables

The primary outcome was a spinal hematoma and the secondary outcomes were spinal cord impingement and SCI. The extent and dimensions of hematomas were recorded. Fracture classification and stability were defined by the AO classification and the three-column concept by Denis, respectively [25, 26]. Polytrauma was defined as at least one injury in addition to a spine fracture [27].

The smallest anterior to posterior width of the dural sac in T2 sagittal images at the level of fracture defined the spinal canal narrowing. Relative spinal canal narrowing was defined as spinal canal anteroposterior width divided by the mean width of the adjacent levels.

Demographics recorded from the medical records included gender, age, and trauma mechanism. Choices for treatment were, conservative or surgical (decompression, anterior, or posterior fixation).

Neurological function was documented using Frankel grades, which were retrospectively reviewed from the medical records at three points in time; paramedics’ reports during the acute phase (Frankel_para_), attending physicians’ evaluation before the decision for either surgical or conservative treatment (Frankel_ward_), and the final evaluation before release to further care-taking units (Frankel_final_) [28]. The change in Frankel grades (Frankel Δ) was calculated by (Frankel_final_—Frankel_ward_)/ Frankel_final_. The definition of Frankel grades is presented in Table 1.

**Table 1:** Frankel grade classification describing neurological function according to Frankel et al. [7]

Grade A	Complete neurological injury with no motor or sensory function below the level of lesion
Grade B	Complete motor paralysis below the level of lesion with possible sensory function present
Grade C	Nonfunctional motor power present below the level of lesion
Grade D	Functionally useful motor power with a possible partial sensory failure below the level of lesion
Grade E	Normal motor and sensory function with possible abnormal reflexes below the level of lesion

## Image analysis

A board-certified radiologist with 1-year experience as a fellow in musculoskeletal and trauma radiology (R.V., reader 1) reviewed all CT scans for spinal ankylosis. A board-certified musculoskeletal and trauma radiologist with 11 years of experience (F.B., reader 2) was blinded to the original reports and results of preselected 125 CT scans with DISH features for the etiology of ankylosis. A board-certified musculoskeletal and trauma radiologist with 16 years of experience (V.H., reader 3) and reader 1 reviewed the 70 MRI scans independently and blinded to one another and the original reports for spinal hematoma, spinal cord impingement, and SCI. Disagreements were settled by consensus. Differentiation of spinal hematomas’ anatomical compartments was defined as being either in the epidural or within the potential subdural space [6].

## Statistical analysis

The correlation between relative spinal canal narrowing and Frankel_ward_ was analyzed using Spearman’s ρ. Associations between variables were analyzed as follows: spinal cord impingement vs. Frankel_ward_ in both surgically and conservatively treated patients (Mann–Whitney U-test), the presence of spinal hematoma in patients treated with decompression (spinal hematoma indicated as a dichotomic ordinal value) vs. Frankel Δ (Mann–Whitney U-test). Patients were divided into four groups: surgically treated patients with cervical (group 1, 26 patients), and thoracolumbar (group 2, 19 patients) fractures; and conservatively treated patients with cervical (group 3, 14 patients), and thoracolumbar (group 4, 11 patients) fractures. For a longitudinal comparison between Frankel_para_ and Frankel_ward_ (test 1) and Frankel_ward_ vs. Frankel_final_ (test 2), we used the Wilcoxon signed-rank test. The abovementioned tests are shown in Table 2. Interobserver agreement between readers 1 and 2 for etiology of ankylosis, and between readers 1 and 3 for MRI findings was calculated by Cohen’s κ [29]. *p* values < 0.05 were considered statistically significant. Strength for interobserver agreement was interpreted as follows: values less than 0, no agreement; 0–20, slight agreement; 0.21–40, fair agreement; 0.41–0.60, moderate agreement; 0.61–0.80, substantial agreement; and 0.81–1, almost perfect agreement. All statistical analyses were performed using SPSS v. 25 (IBM Corp.).

**Table 2:** Results of the statistical tests used in the study for the neurological function of spinal injury in patients with spinal ankylosis by diffuse idiopathic skeletal hyperostosis. Group 1 = surgically treated patients with a cervical spine injury, group 2 = surgically treated patients with a thoracolumbar spine injury, group 3 = conservatively treated patients with a cervical spine injury, group 4 = conservatively treated patients with a thoracolumbar spine injury

Test variables			Statistical tests and results	
Relative spinal canal narrowing vs. Frankel_ward_			Spearman’s ρ 0.519, *p* < 0.001	
Spinal cord impingement in surgically treated patients vs. Frankel_ward_			Independent samples Mann–Whitney U test, *p* = 0.004	
Spinal cord impingement in conservatively treated patients vs. Frankel_ward_			Independent samples Mann–Whitney U test, *p* < 0.001	
Presence of spinal hematoma in patients treated with decompression vs. Frankel Δ			Independent samples Mann–Whitney U test, *p* = 0.53	
	Test 1 variables	Statistical tests and results	Test 2 variables	Statistical tests and results
Group 1	Frankel_para_ vs. Frankel_ward_	Related samples Wilcoxon signed rank test, *p* = 0.129	Frankel_ward_ vs. Frankel_final_	Related samples Wilcoxon signed rank test, *p* < 0.001
Group 2	Frankel_para_ vs. Frankel_ward_	Related samples Wilcoxon signed rank test, *p* = 0.024	Frankel_ward_ vs. Frankel_final_	Related samples Wilcoxon signed rank test, *p* = 0.004
Group 3	Frankel_para_ vs. Frankel_ward_	Related samples Wilcoxon signed rank test, *p* = 0.257	Frankel_ward_ vs. Frankel_final_	Related samples Wilcoxon signed rank test, *p* = 0.063
Group 4	Frankel_para_ vs. Frankel_ward_	Related samples Wilcoxon signed rank test, *p* = 0.317	Frankel_ward_ vs. Frankel_final_	Related samples Wilcoxon signed rank test,* p* = 1

## Results

### Patients and outcomes

**Fig. 2:**
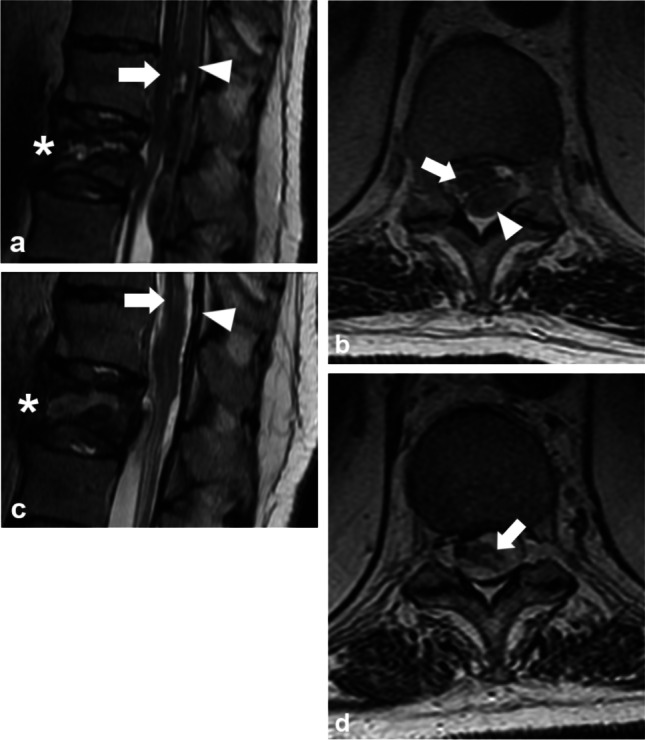
A 70-year-old patient after GLF was admitted to the local health care center with low back pain. During 5 days, the pain increased to the point of restricting movement. An MRI scan 11 days after the injury revealed a transverse fracture through the body of L1 (asterisk) with an epidural hematoma (arrowhead) causing impingement of the spinal cord (arrow) on sagittal T2WI (**a**). The epidural hematoma (arrowhead) causes impingement of the spinal cord (arrow) on transverse T2WI (**b**). After the refusal of surgical treatment, radicular pain to the lower limbs continued, and an MRI scan after 18 days showed a non-ossified fracture of the first lumbar vertebra (asterisk) and faint focal signal intensity in the spinal cord indicating spinal cord injury (arrow) on sagittal T2WI. The epidural hematoma, causing spinal cord impingement, had mainly resorbed leaving uneven traces of hemosiderin (arrowhead) (**c**). On transverse T2WI, at the level of the Th12, the spinal cord injury is distinctly visible (arrow) (**d**). MRI = magnetic resonance imaging, GLF = ground-level fall, L1 = first lumbar vertebra, Th12 = twelfth thoracic vertebra

**Fig. 3:**
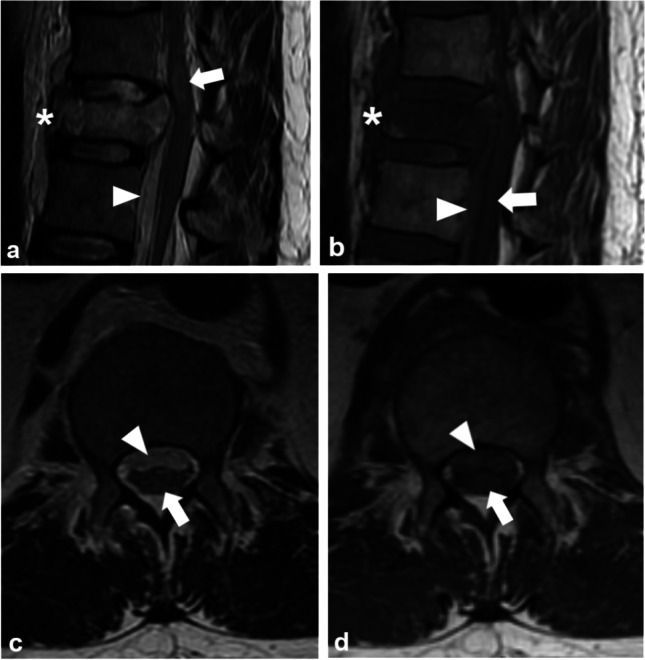
A 66-year-old patient fell three meters off a ladder on his stomach and was unable to move his lower limbs. A sagittal T2WI from an MRI scan revealed a spinal cord injury (arrow) caused by a burst fracture of Th12 (asterisk). Anteriorly to the spinal cord is a hyperintense spinal hematoma (arrowhead) visible in the subdural space (**a**). On sagittal T1WI, the subdural hematoma (arrowhead) appears hyperintense compared to the spinal cord (arrow). The burst fracture is shown as a low-intensity area on T1WI representing multiple fracture lines and oedema across the vertebral body (asterisk) (**b**). On transverse T2WI, the subdural hematoma (arrowhead) is contained within the dural sac and pressed against the spinal cord (arrow) (**c**). On transverse T1WI, the subdural hematoma (arrowhead) shows a higher intensity compared to the posteriorly located spinal cord (arrow) (**d**). MRI = magnetic resonance imaging, Th12 = twelfth thoracic vertebra

Of the 70 patients (54 men, median age 73, interquartile range (IQR) 66–81) with DISH and spinal fracture, 37 (53%) had spinal hematoma, 47 (67%) had spinal cord impingement, and 43 (61%) had SCI. Of the 37 spinal hematomas, 34 were SEHs (Fig. 2) and 3 were SSHs (Fig. 3) leading to incidences of 49% and 4%, respectively. Of the 37 patients with a spinal fracture and a spinal hematoma, 30 (81%) had spinal cord impingement. Of the 43 patients with SCI, 7 (16%) had intramedullary hemorrhage. One conservatively treated patient had spinal cord impingement caused by SEH which progressed to SCI (Fig. 2).

**Fig. 4:**
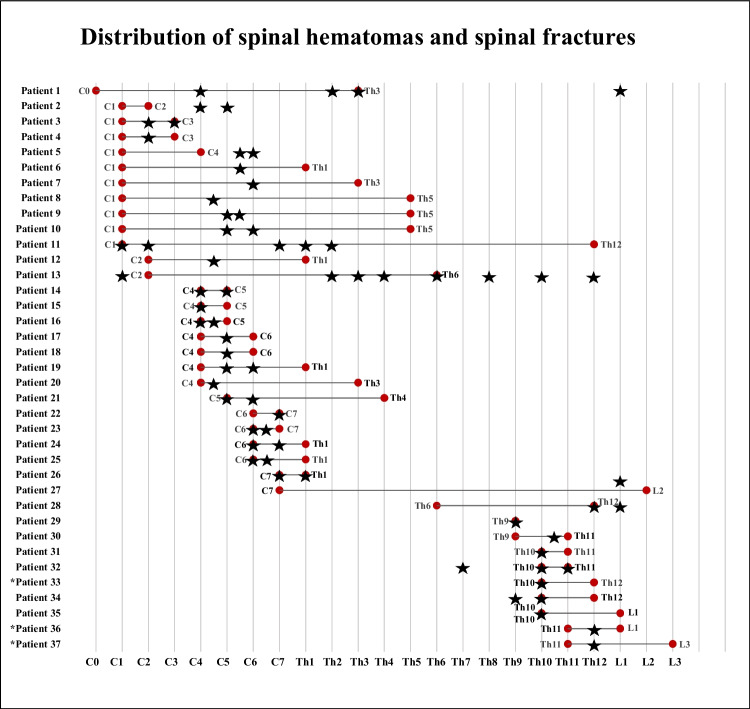
Distribution of spinal hematomas and the related spinal fractures in relation to vertebral levels for 37 patients. The extent of hematomas is shown as lines with red dots representing the borders. Fracture levels are marked as black stars. Patients are not in chronological order. The three patients marked with asterisks had spinal subdural hematomas

The median craniocaudal extent of hematomas was 7 cm (IQR 3,6–15), median thickness 0.5 cm (IQR 0.5–0.7), and median width 2.1 cm (IQR 1.8–2.4). The distribution and extent of spinal SEHs and SSHs in relation to fractures are displayed in Fig. 4.

## Trauma mechanisms, injuries, and fractures

Of the 70 patients with spinal ankylosis by DISH, the most common trauma mechanism was a GLF in 48 (69%) patients, followed by motor vehicle accidents in ten (14%) and falls from or over two meters height with nine (13%) patients. Three patients (4%) fell on a bicycle. One patient fell on ground level after a body check in an ice hockey match.

Twenty-one patients had polytrauma of whom five had a spinal fracture and simultaneous intracranial injury or fractures of the skull or facial bones. Five patients had a blunt cerebrovascular injury in addition to cervical spine fracture.

Seventy patients had a total of 153 fractures, of which 70 were considered unstable in 66 patients (94%). Of 80 type B fractures, 60 (75%) ran transversally through the vertebral body, of which 20 (25%) continued into the adjacent intervertebral disc. Twenty-one (14%) fractures ran completely through the intervertebral disc. Of 27 type A fractures, 19 (70%) were compression fractures and 8 (30%) were burst fractures. Sixteen patients had 19 (12%) fractured spinous processes. Two patients had type C fractures of the first cervical vertebra, aka Jefferson’s fracture. Five patients had type A fractures of the second cervical vertebra and three had type C fractures, aka Hangman’s fracture.

**Fig. 5:**
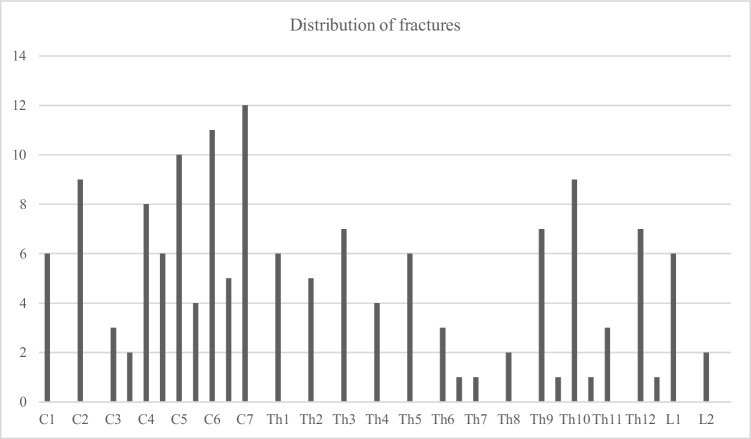
Distribution of spinal fractures in relation to vertebral levels

The number of fractures in relation to vertebral levels peaked at the craniocervical, cervicothoracic, and thoracolumbar junctions (Fig. 5).

## Treatment groups

Of 45 patients (64%) treated surgically, 41 (91%) had posterior fixation. Of four patients with unstable cervical spine fractures, two were treated with both posterior and anterior fixation and two with only anterior fixation. A total of 29 patients with posterior fixation and one with anterior and posterior fixation received decompression. Twenty patients with posterior fixation and spinal hematoma received decompression and ten patients with posterior fixation and spinal hematoma did not undergo decompression. Of the 25 patients (36%) treated conservatively, ten had spinal hematomas.

## Statistical analysis

The relative spinal canal narrowing correlated with Frankel_ward_ (Spearman’s ρ 0.519, *p* < 0.001). Spinal cord impingement was associated with Frankel_ward_ in surgically (*p* = 0.004) and conservatively (*p* < 0.001) treated patients (Mann–Whitney U).

The presence of spinal hematoma in patients treated with decompression and the change in Frankel grading after decompression (Frankel Δ) showed no significant association (*p* = 0.53; Mann–Whitney U). Of the 30 patients with decompression and posterior fixation (one patient without spinal hematoma had both anterior and posterior fixation), 20 had a spinal hematoma.

The Wilcoxon signed-rank test showed significance in Group 1 for Frankel_ward_ vs. Frankel_final_ (*p* < 0.001), and in group 2 for both; Frankel_para_ vs. Frankel_ward_ (*p* = 0.024) and Frankel_ward_ vs. Frankel_final_ (*p* = 0.004).

Interobserver agreement between Readers 1 and 2 for the etiology of ankylosis was moderate (κ = 0.570, *p* < 0.001). Interobserver agreement between readers 1 and 3 for MRI findings was moderate (*κ* = 0.602) for spinal hematoma, substantial (*κ* = 0.683) for SCI, and substantial (*κ* = 0.664) for spinal cord impingement (*p* < 0.001).

## Discussion

Caron et al. presented the first large-scale series representing both ankylosing spondylitis and DISH after spinal trauma and found SEH in five of the 74 patients with DISH and an incidence of 5% [18]. In a retrospective review, after a search from a database for DISH, Bransford et al. found three SEHs by evaluating the medical records of 33 patients for an incidence of 9% [19]. Balling and Wenkbach found one patient with neurological deficits to have SEH for an incidence of 0.5% in a series of 20 DISH patients imaged by CTs and radiographs [20]. In 2017, Okada et al. made no mentions of spinal hematomas from 32 MRI scans of 42 patients with DISH [21]. Two years later, the same group studied a larger retrospective multicenter series of 285 patients with post-traumatic DISH patients of whom 203 had MRI scans [23]. Examination of medical charts revealed 22 SEHs reaching an incidence of 10.8%. In a retrospective review of 134 patients found from a data registry search by ICD-9 and ICD-10 codes, Teunissen et al. showed 5 SEH in 26 DISH patients for a remarkably high incidence of 19.2% [22]. After a review of 147 CT scans of post-traumatic DISH patients with low-energy trauma, imaged with 9 MRI scans, Lantsman et al. made no mentions of SEHs [4].

In the largest and most recent retrospective multicenter study of 160 DISH patients, Shah et al. found 160 patients imaged by CT using a keyword search from radiological studies [24]. Of those, 94 had MRI scans from which the involvement of the posterior ligamentous complex was reviewed, and SEH was found with an incidence of 5%.

Lee described a spinal subarachnoideal hematoma in a single case report of a post-traumatic DISH patient [14]. To our knowledge, there are no previous studies published examining SSH in post-traumatic DISH patients.

The current study shows an incidence of 49% and 4% for SEH and SSH, respectively, in post-traumatic patients with spinal fractures and ankylosis caused by DISH, showing that SEH is a common complication, and SSH is less common.

The most common trauma mechanism was GLF in 69% in accordance with previous studies’ results [19, 21, 22, 24], which is attributable to both age and mobility restrictions in this demographic. The mobility of the craniocervical junction in relation to the rigid upper parts of the thoracic spine produces extension forces in the lower parts of the cervical spine when falling forwards or backwards which is highlighted in Fig. 5. A fracture in the cervical spine is more likely to cause a SCI than a fracture in the inherently more stable thoracic spine, which is supported by the ribs and the surrounding apophyseal joints [22]. With increasing falling height, the distribution of fractured vertebrae in the current study would have likely shown a higher peak around the thoracolumbar junction (Fig. 5) [20, 23, 30].

Previous studies have shown a larger share of fractures running through the intervertebral discs in post-traumatic DISH patients [19–21]. In recent publications as well as the current study, most of the fractures were found starting from the anterior parts of the vertebral body and running through it which seems to be the dominant fracture pattern in patients with ankylosis from DISH [23, 24]. This pattern is likely due to the protective effect of the DISH syndesmophytes at the level of the intervertebral disc and becomes more common in the elderly [20].

The correlation between the relative spinal canal narrowing and Frankel_ward_, and the association between spinal cord impingement and Frankel_ward_ suggests that spinal hematoma impairs neurological function within hours as previously reported [8]. However, this study found no differences in the improvement of neurology between spinal hematoma patients who had posterior fixation and those with both posterior fixation and decompression. This suggests that spinal hematoma plays a less significant role in the development of neurological deficits than bony fragments and fibrotic or calcified spinal ligaments. Nevertheless, when the spinal hematoma is the only cause of spinal cord impingement and treatment choices deter from decompression, SCI may still occur (Fig. 2).

Insignificant change in test 1 and a statistical difference in test 2 for improvement of Frankel grade in group 1 suggests that a cervical spine fracture tends to cause immediate spinal cord impingement or SCI with immediate neurological symptoms sufficiently severe to mandate surgical treatment (Table 2). The improvement from Frankel_ward_ to Frankel_final_ scores shows that these patients benefit from surgical treatment. A nondislocated fracture of the thoracolumbar spine is more likely to be stable and therefore might allow residual mobility before the onset of neurological impairment, as evidenced by differences between Frankel_para_ and. Frankel_ward_ in group 2. The difference between Frankel_ward_ and Frankel_final_ shows that these patients also benefit from surgical intervention. As expected, Frankel grades did not degrade between Frankel_para_ and Frankel_ward_ in patients of group 3 and group 4 selected for conservative treatment, which can be explained by either minor injuries, or patients’ overall condition preventing surgical treatment.

**Fig. 6:**
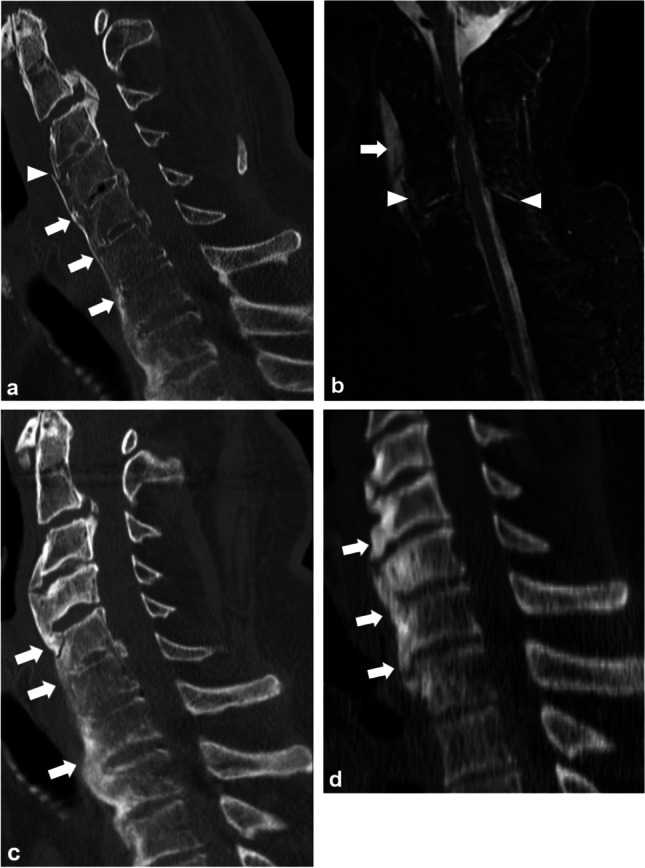
A 60-year-old male after GLF to his lower back without direct impact to the head or the neck. A sagittal CT image shows anteriorly bridging syndesmophytes (arrows) with features of both ankylosing spondylitis and DISH. A small fracture (arrowhead) is evident at the calcified anterior longitudinal ligament of the intervertebral space between the third and the fourth cervical vertebra (**a**). The fracture (arrowheads) running through the anterior body of the fourth cervical vertebra and along the upper endplate of the fifth cervical vertebra continuing to the ligamentum flava and the interspinous ligament is more distinct on sagittal STIR, which also reveals a prevertebral hematoma (arrow) (**b**). A sagittal CT image taken 6 years prior to injury shows more prominent bridging syndesmophytes (arrows) at the anterior parts of the intervertebral discs (**c**). A sagittal CT reformat from lower-resolution images taken 16 years prior to injury shows recognizable syndesmophytes (arrows) indicative of DISH (**d**). MRI = magnetic resonance imaging, STIR = short tau inversion recovery, GLF = ground level fall

The retrospective study setup and the small sample size were obvious limitations of this study. All patients suffering from spinal trauma with CT imaging did not receive an MRI scan and therefore the true incidence of spinal hematomas remains unclear. The overlapping features of SpA and DISH affected the inter-rater agreement in distinguishing both entities from each other (Fig. 6) [22]. Due to our institution being a level-one trauma center, patients tend to have more acute and serious injuries on average which may lead to selection bias. Additionally, not all regional hospitals offer emergency MRI scans outside of business hours, which is why patients with neurological deficits will often be referred to a higher-level center. Although previous studies exceed our sample size, to our knowledge, the current study is the first to discuss spinal hematoma, spinal cord impingement, and SCI in post-traumatic patients with spinal ankylosis by DISH.

In conclusion, SEH is a common complication in post-traumatic patients with spinal ankylosis by DISH and tends to occur following low-energy trauma. The spinal hematoma tends to evade the bony fragments, but a minority of patients (1%) will develop SCI caused by mass effect from the expanding hematoma. Type B spinal fractures through the vertebral body are the most common pattern leading to unstable injury.

## Abbreviations

CT: Computed tomography
DISH: Diffuse idiopathic skeletal hyperostosis
GLF: Ground-level fall
IQR: Interquartile range
MRI: Magnetic resonance imaging
SCI: Spinal cord injury
SEH: Spinal epidural hematoma
SpA: Seronegative spondylarthropathy
SSH: Spinal subdural hematoma
STIR: Short tau inversion recovery

## Declarations

### Guarantor

The scientific guarantor of this publication is Frank Bensch.

### Statistics and biometry

Olavi Koivisto kindly provided statistical advice for this manuscript.

### Methodology

- retrospective study
- observational study
- study performed at one institution
